# Development of the Teeth, with Their Arterial and Nervous Supply

**Published:** 1881-05

**Authors:** Jennie C. Kollock


### THE DENTAL REGISTER.

Vol. XXXV.] MAY, 1881. [No. 5.

### DEVELOPMENT OF THE TEETH, WITH THEIR ARTERIAL AND NERVOUS SUPPLY.

JENNIE C. KOLLOCK, D. D. S.

Every organ of the human body whether of greater or less dimensions or importance, has its function to perform; and not one is capable of performing this work, without the aid of another. The smallest muscle, artery, vein or capillary, although independent of other organs in their local work, which in a state of health is always faithfully performed, yet without the aid of other organs and tissues from which to gather and return, strength, nourishment and force, must of themselves be deprived of all action; and these as well as greater organs, or those whose functions seem of greater importance, are dependent upon vital phenomena, for their strength utility or adaptation. The muscles act only in their normal condition and place, being connected with the bones cartilages, ligaments or skin, directly or indirectly through fibrous structure. The arteries would be useless vessels if the heart the great propeller of the human blood, failed in performing its wonderful work.

If the coursing of blood through arteries ceased, veins and capillaries would become useless, and the motor nerves which tell us how and when to act, as well as the sensor through which all sensations of thought, feeling or sentiment are transmitted, are but children of the brain, that grand centre of all human action and emotion.

Thus we see each and every organ dependent upon and wonderfully and beautifully adapted to another, making up one grand whole, a human body, the greatest masterpiece in earthly existence. Among the most important of the organs of which we have been speaking, are the teeth, which perform many and varied functions; the principal one of which is the mastication of the food, making them the first of the great organs of digestion; again they are among the organs of speech, without which many words could not be uttered fully, distinctly nor at all correctly; they also aid very materially in giving form to the face, and expression to the features.

The human subject is provided with two sets of teeth, which make their appearance at different periods of life.

The first set which appears in childhood are called the temporary, deciduous or the milk teeth. The second set which also appears at an early age, continuing until late in life are called permanent. The first set of teeth are twenty in number, and are developed according to Goodsir, in the following manner:

"Six weeks after conception the upper jaw of the embryo presents two semi-circular folds around its circumference; the most external one being the true lip, the internal being the true palate, while between these is found a deep groove lined by the common mucous membrane of the mouth, a little later than this a ridge is developed from the floor of this groove. This is the rudiment of the external alveolus, and the arrangements of the appearances from without inwards, at this period is the following: most externally and forming the boundary of the mouth is the lip, next we find a deep groove which separates the lip from the future jaw, then comes the external alveolar ridge; fourthly, another groove in which the germs of the teeth are developed, the primitive dental groove; fifthly a rudiment of the internal alveolar ridge, and sixthly the rudiment of the internal future palate bounding the whole internally.

At the seventh week the germ of the first deciduous molar of the upper jaw has made its appearance, in the form of a simple free granular papilla of the mucus membrane, projecting from the floor of the primitive dental groove.

At the eighth week the papilla of the cuspid tooth is developed; at the ninth week, the papilla of the four incisors

(the middle preceding the lateral) appear; at the tenth week, the papilla of the second molar is seen, behind the anterior molar in the primitive dental groove."

So that we find as early as the tenth week of intra-uterine existence, the germs of all the deciduous teeth of the upper jaw.

It has been ascertained by similiar investigation, that those of the lower jaw are a little more tardy in making their appearance, the papilla of the first molar being merely a slight bulging at the seventh week, and the tenth papilla is not apparent until the eleventh week.

Goodsir says farther that from the eighth week the primitive dental groove becomes contracted, before and behind the first deciduous molar, and lamina of the mucus membrane are developed around the other papilla, which increase in growth and enclose the papilla in follicles with open mouths.

At the tenth week the follicle of the first molar is completed, then that of the cuspid, during the eleventh and twelfth weeks the follicles of the incisors succeed, and at the thirteenth week, the follicle of the posterior deciduous molar is found.

During the thirteenth week the papilla undergoes a change of form and assumes the shape of the teeth they are intended to represent; at the same time small membranous processes are developed from the mouths of the follicles, these processes are intended to serve the purpose of opercula to the follicles, corresponding in shape to the form of the crowns of the appertaining teeth.

To the follicles of the incisor teeth there are two opercula, to the cuspids three, and to the molars a number corresponding to the number of their tubercles either four or five.

During the fourteenth and fifteenth weeks, the opercula have completely closed the follicles, so as to convert them into dental sacs, and at the same time, the papilla have become pulps.

The portion of the groove which contains the dental sacs of the deciduous teeth is now closed in, and the remaining portion, that which is nearest the gum, is still left open and is called the secondary dental grove, giving space for the development of the permanent teeth, with the exception of the anterior molars. By a process fully as complete and wonderful as the development of the deciduous, the permanent are formed and erupted.

First we have papilla of mucus membrane, which in these are called "cavities of reserve," they at first lie near the surface of the gum, but finally recede from there, and lie at the inner side and in close contact with the dental sacs of the deciduous teeth.

At about the fifth month the anterior of these cavities of reserve dilate at their distal extremity, and a papilla projects into their fundus which constitutes the rudiment of the germ of the permanent tooth, as the formation and growth of these teeth are almost precisely similar to those of the upper, a farther description of the same seems hardly necessary.

The process of dentition is divided according to Goodsir, into three stages. First, follicular; second, saccular; third, eruptive. The first or follicular, he makes to include all the changes which take place from the first appearance of the dental groove and papilla, to the closure of their follicles; thus occupying a period extending from the sixth week to the fourth or fifth month of intra-uterine existence.

The second, or saccular stage, comprises the period when the follicles are closed sacs and the included papilla are pulps. The third or eruptive stage includes the completion of the teeth, the eruption and shedding of the temporary set, the eruption of the permanent and the necessary changes in the alveolar process, extending from the seventh month, until the twenty-first year.

The anterior permanent molar is thought by anatomists to be the most remarkable tooth in the human subject, as it forms a transition between the deciduous and the permanent set. If considered anatomically, that is in its development from the primitive dental groove by a papilla and follicle, it is certainly a deciduous tooth; if physiologically, as the most efficient grinder in the adult mouth, we must consider it a permanent tooth.

Immediately after the closing of the dental follicles by their opercular, the pulps (which consist of jelly-like substance, being highly vascular and having a nervous supply) become moulded into the forms of the future teeth, and the basis of the molars divided into two or three portions representing the future roots.

In the dental sac enclosing the teeth or their rudiments, we find two layers an internal vascular, and an external which is cellulo-fibrous in structure, and now by a curious and interesting process, layers of dentine are formed and deposited upon the pulp, first upon its upper part or crown, then upon the sides, forming a sort of cap, and covering the exposed surface; layers of enamel are next formed and deposited over the surface, and in this way the tooth becomes complete.

The periods for the eruption of the teeth are extremely irregular, but the average time for the deciduous are as follows: At the seventh month, two middle incisors; ninth month, two lateral incisors; twelfth month, first molar; eighteenth month, cuspids; twenty-fourth month, two last molars.

And the average periods for the eruption of the permanent, are six and a half years, first molar; seventh year, two middle incisors; eighth year, two lateral incisors; tenth year, second bicuspids; eleventh to the twelfth year, cuspids; twelfth to the thirteenth year, second molars; seventeenth to twenty-first year, last molars.

A tooth is composed of three parts, namely: crown, neck and root; the crown is that part projecting above the gum; the neck, the constricted part at the base of the crown, and the root is that part contained within the alveolus.

Teeth vary in shape to suit the different purposes for which they were designed; the incisors and cuspids are for cutting purposes, and have sharp beveled crowns, with single cusps, while the biscupids and molars, used for grinding the food have broad crowns with from four to five cusps.

The dentine forms the principal part of the crown of the tooth; it consists of minute tapering and branching fibers, called tubuli, imbedded in a homogeneous mass of inter-fibrous substance, these tubuli have a radiating course from the pulp toward the periphery of the tooth; and they terminate either in clubbed extremities, in loops, or in very minute points.

The enamel forms a crust or layer over the entire crown, it is the hardest substance in the body, is composed of minute hexagonal rods, which rest by one extremity against the dentine, the other radiating outward to the enamel cuticle.

The cementum, or crusta petrosa covers the root from the neck, where it overlaps the enamel, to the apex. It is analogous to bone in its structure; is evenly but sparsely distributed, but increases in thickness as age advances.

The pulp occupies the cavity in the crown and root of the tooth, it is a soft, vascular, and highly sensitive substance of a redish gray color; it gives nourishment, support and sensibility to the tooth. According to some authors, fibers from the pulp pass out in all directions through the dental tubuli, by others that a fluid only is contained in the tubuli.

This substance whether it be fibers or only nerve fluid, nourishes the dentine and gives to it sensibility.

The chemical composition of the pulp is Oxygen, Hydrogen, Nitrogen and Carbon.

That of the dentine and enamel, phosphate and carbonate of lime, with a trace of fluoride of calcium, phosphate of magnesia, and other salts; besides this, the dentine contains 28 per cent. of animal matter, while the enamel being much the harder structure contains but 2 to 4 per cent. of the same. The roots of the teeth are covered with a membranous substance, called the periosteum, which is reflected on to the socket or alveolar process. It is very highly endowed with vitality, being extremely vascular and gives nourishment to the root so that it retains considerable vitality, even after the death of the pulp.

The nerves supplied to the teeth are branches from the trifacial or fifth, the largest and most important of all the cranial nerves; these branches are the superior and inferior maxillary; the teeth in the upper jaw supplied from the former. Just before it enters the infra-orbital canal it gives off a branch called the posterior dental, this passes from behind forward in the substance of the bone of upper jaw, and joins opposite the canine fossa with the anterior dental; numerous filaments are given off from the lower border of this nerve which form a minute plexus in the outer wall of the superior maxillary bone, immediately above the alveolus; from this plexus filaments are distributed to the pulps of the molar and second bicuspid teeth.

The anterior dental is of large size and is given off from the superior maxillary nerve just before its exit from the infra-orbital foramen; it enters a special canal in the anterior wall of the antrum and anastomoses with the posterior dental; from this nerve some filaments are distributed to the incisor, cuspid and first bicuspid teeth.

The teeth in the lower jaw are supplied from the inferior maxillary nerve, by a branch called the inferior dental, it passes down with the inferior dental artery, at first beneath the exteral pterygoid muscle, and then between the internal lateral ligament and the ramus of the jaw to the dental foramen, it then passes forwards in the dental canal in the inferior maxillary bone, lying beneath the teeth as far as the mental foramen, where it divides into two terminal branches the incisor and mental, the incisor is continued onwards within the bone to the middle line, and supplies the cuspid and incisor teeth, the mental branch emerges on the face at the dental foramen and supplies the structures composing the chin.

The arterial supply to the teeth is from the internal maxillary, it being the larger of the two terminal branches of the external carotid artery. This artery is divided by anatomists into three portions, according to the parts of the face over which it traverses. The first portion is called the maxillary; it passes forwards and inwards between the ramus of the inferior maxillary jaw, and the internal lateral ligament crossing the inferior dental nerve. In this situation it gives off several branches among which is the inferior dental, this branch passes downwards with the dental nerve to the foramen on the inner side of the ramus of the jaw; it is continued along the dental canal in the substance of the bone together with the nerve, and opposite the first bicuspid tooth, divides into two branches called the incisor and mental, the former is continued forward beneath the incisor teeth, as far as the median line or symphysis, here it anastomoses with its fellow of the opposite side. The mental branch emerges upon the face with the nerve at the mental foramen, supplying the muscles and integument of the chin.

The dental and incisor arteries and nerves, during their passage through the dental canal, give off minute branches corresponding in number to the roots of the teeth beneath which they pass; these branches enter the small apertures at the extremities of the roots and in this way supply the teeth.

The third or spheno-maxillary portion is the next part of the internal maxillary, which gives off branches to the teeth. Here it approaches the superior maxillary bone, and enters the spheno maxillary fossa, in which situation it gives off many branches, among which is the alveolar and infra orbital, each of which in turn, gives off branches supplying the teeth.

The alveolar is given off just as the trunk of the internal maxillary is passing into the spheno maxillary fossa, it then passes down upon the tuberosity of the superior maxillary bone and divides into numerous branches. One the superior dental being larger than the rest, supplies the molar and bicuspid teeth, its branches entering the foramina in the alveolar process.

The infra-orbital, is thought to be from its direction, the continuation of the trunk of the internal maxillary; it arises from that vessel by a common trunk with the alveolar, and traverses the infra-orbital canal with superior maxillary nerve. While it is contained in the canal, it gives off branches which descend through canals in the bone, thus giving supply to front teeth of upper jaw.

